# Preference-aligned fertility management among married adolescent girls in Northern Nigeria: assessing a new measure of contraceptive autonomy

**DOI:** 10.1136/bmjgh-2023-013902

**Published:** 2024-05-16

**Authors:** Claire W Rothschild, Alhaji Bulama, Roselyn Odeh, Salome Chika-Igbokwe, Julius Njogu, Katherine Tumlinson, Abednego Musau

**Affiliations:** 1 Sexual and Reproductive Health, Population Services International, Washington, District of Columbia, USA; 2 Society for Family Health Nigeria, Abuja, Nigeria; 3 Sexual and Reproductive Health, Population Services International, Nairobi, Kenya; 4 University of North Carolina, Chapel Hill, North Carolina, USA; 5 Carolina Population Center, University of North Carolina, Chapel Hill, North Carolina, USA

**Keywords:** cohort study, public health, health systems

## Abstract

**Introduction:**

Universal access to sexual and reproductive healthcare—including family planning (FP)—is a global priority, yet there is no standard outcome measure to evaluate rights-based FP programme performance at the regional, national or global levels.

**Methods:**

We collected a modified version of preference-aligned fertility management (PFM), a newly proposed rights-based FP outcome measure which we operationalised as concordance between an individual’s desired and actual current contraceptive use. We also constructed a modified version (satisfaction-adjusted PFM) that reclassified current contraceptive users who wanted to use contraception but who were dissatisfied with their method as not having PFM. Our analysis used data collected 3.5 months after contraceptive method initiation within an ongoing prospective cohort of married adolescent girls aged 15–19 years in Northern Nigeria. We described and compared prevalence of contraceptive use and PFM in this population.

**Results:**

Ninety-seven per cent (n=1020/1056) of respondents were practising PFM 3.5 months after initiating modern contraception, while 93% (n=986/1056) were practising satisfaction-adjusted PFM. Among participants not practising satisfaction-adjusted PFM (n=70), most were using contraception but did not want to be (n=30/70, 43%) or wanted to use contraception but were dissatisfied with their method (n=34/70, 49%), while the remaining 9% (n=6/70) wanted but were not currently using contraception.

**Conclusion:**

PFM captured meaningful discordance between contraceptive use desires and behaviours in this cohort of married Nigerian adolescent girls. Observed discordance in both directions provides actionable insights for intervention. PFM is a promising rights-focused FP outcome measure that warrants future field-testing in programmatic and population-based research.

**WHAT IS ALREADY KNOWN ON THIS TOPIC**

- Universal access to quality sexual and reproductive healthcare is currently measured using contraceptive prevalence, a measure with fails to directly ask individuals whether they wish to use contraception.
- New, rights-based family planning (FP) outcome measures are urgently needed to refocus monitoring and evaluation of FP programmes on fulfilment of rights rather than on contraceptive utilisation.

**WHAT THIS STUDY ADDS**

- This study is among the first to capture a newly proposed rights-based FP measure, preference-aligned fertility management (PFM).
- In a cohort of married adolescent girls recently initiating modern contraception, we found that 7% reported contraceptive use that did not match their desired preferences.
- Among these, nearly all (91%) were either using contraception but did not wish to be or were dissatisfied with their current contraceptive method.

**HOW THIS STUDY MIGHT AFFECT RESEARCH, PRACTICE OR POLICY**

- PFM is a promising measure for evaluating the success of rights-based FP programmes.
- It is conceptually simple, requires few additional data collection requirements in addition to standard surveys and provides actionable insight for programme implementers and policymakers to address constraints to self-perceived contraceptive autonomy.

## Introduction

Universal access to sexual and reproductive healthcare—including family planning (FP)—is a global priority codified in Sustainable Development Goal Target 3.7, yet there is currently no standard approach to assessing rights-based FP programme outcomes. Instead, FP programme performance and global monitoring systems have for decades relied on a set of outcome measures that primarily capture modern contraceptive utilisation. At their core, the commonality of the current guard of FP outcome measures is that they centre modern contraceptive utilisation and discontinuation/non-use as uniformly ‘good’ and ‘bad’ outcomes, respectively, without consideration of individual preference.^1^

The shortcomings of modern contraceptive prevalence (mCP) and ‘need’-based FP measures^2^ are more than academic: they can lead to misalignment between FP programme and policy aims on one hand, and evidence-based decision-making, resource allocation and programme implementation on the other.^3^ Specifically, although ‘unmet need’ is often interpreted as a lack of access to contraception, the vast majority of ‘unmet need’ is due to lack of demand.^4^ Contraceptive utilisation-based measures can also incentivise coercion even in the absence of numeric targets or quotas.^5 6^ mCP and its ‘need’-based derivatives are measures of point prevalence— a product of both the *incidence* and the *duration* of contraceptive use. As such, programmes that are evaluated using these measures are incentivised to maximise uptake of *any* contraceptive method, and—more subtly—to maximise uptake of specific methods with the longest average duration of use. Even as the FP field has acknowledged the problems of tiered-effectiveness counselling,^7^ there has been less recognition of the ways in which performance measurement using mCP continues to reinforce ‘long-acting reversible contraceptive (LARC) first’ programming. Finally, mCP and ‘need’-based measures may be particularly insensitive for measuring the success of FP programmes that target adolescents and young people. Continuous contraceptive use may not be a relevant or desirable outcome for many adolescents and youth, who are often more likely to abstain or have infrequent sex than their older counterparts.^8^ As a result, there is an urgent need for measures that centre autonomy and fulfilment of rights to measure the success of FP programmes, including those specifically targeting adolescents and young people.

There is increasing momentum in the global FP field to develop such rights-based measures.^9^ Drawing from the rich scholarship and activism of the reproductive justice movement led by black feminists in the USA,^10 11^ many are calling for person-centred indicators that prioritise reproductive justice and autonomy.^1 12 13^ Senderowicz has proposed ‘contraceptive autonomy’ as a novel measure of rights-based FP programming that, ‘redefines success in FP as concordance between what a person wants and what they have, regardless of contraceptive use status’.^1^ Drawing from Senderowicz’s core conceptualisation of contraceptive autonomy, Holt *et al* proposed an alternative measure called ‘preference-aligned fertility management’ (PFM).^14^ As proposed by Holt *et al*, PFM measures concordance between women’s stated desire to currently use a specific method(s) of contraception and their self-reported current use of that method(s).^14^ PFM is attractive as a novel measure due to its parsimony: measurement of PFM requires the addition of only a few new questions to standard contraceptive questionnaires.

We fielded two modified versions of the novel PFM measure within an ongoing prospective cohort of married adolescent girls initiating modern contraception in Northern Nigeria in order to describe the prevalence and utility of the measure as an alternative to traditional contraceptive-use focused end points. Our analysis uses data collected from the cohort at enrolment (which occurred on the date of facility-based modern contraceptive initiation) and at the first follow-up survey 3.5 months later. Our primary aim was to assess PFM in this population in order to describe concordance between current and desired contraceptive use and to identify individual sociodemographic and contraceptive characteristics associated with PFM. We consider implications of incorporating method dissatisfaction as another possible dimension of discordance between contraceptive preferences and behaviours.

## Methods

### Study setting

The analyses presented in this paper use data collected as part of an ongoing prospective cohort study conducted in Northern Nigeria. The study is being conducted within the Igabi, Zaria, Chikun and Sabon Gari local government areas (LGAs) of Kaduna State and the Karu and Doma LGAs of Nasarawa State. Participants comprise married adolescent girls newly initiating a modern contraceptive method within 15 public health facilities in these study sites at the point of study enrolment. These 15 health facilities were selected due to their participation in the Matasa Matan Arewa (MMA) programme, which is implemented by Society for Family Health-Nigeria in collaboration with the Nigerian government as part of the multicountry Adolescents 360 (A360) Project led by Population Services International.^15^ The MMA programme aims to expand access to voluntary FP services for married adolescent girls in Northern Nigeria by delivering FP services integrated with programming to support economic skills-building and life goal setting. The MMA programme operates within partnering public health facilities to implement a four-pronged intervention package including training and job aids to supporting high-quality, person-centred contraceptive counselling; reminders and ongoing support for method initiators; deployment of a cadre of ‘Big Sistas’, community-based peer educators authorised to provide refills of select short-acting contraceptive methods and community interventions to support male engagement and peer support through interpersonal communication agents, religious leaders and ‘life, family and health’ classes within catchment area communities.

Married adolescent girls were recruited between October and November 2022 while leaving participating health facilities, where they were verbally screened for eligibility within a private location at the facility. Girls were eligible for study participation if they were aged 15–19 years, currently married, newly initiating a method of modern contraception (including first time users, users with history of contraceptive use but no current use on arrival at the facility and those switching from one method type to another), received the contraceptive method via a facility supported by the MMA programme, willing to participate in baseline and follow-up assessments and not intending to migrate outside of the study area within the next 1 year. Girls who were seeking services for method continuation (ie, to obtain a refill of a short-acting contraceptive method) were not eligible to participate.

### Data collection

Trained study enumerators administered an in-person baseline survey at the point of study enrolment. The first follow-up survey was conducted between February and March 2023, 3.5 months after method initiation (the date of study enrolment). This time period is based on the 15-week effectiveness window for injectable methods stipulated by the US Centers for Disease Control, in order to ensure that contacting participants for the follow-up survey did not inadvertently introduce upwards bias to estimates of contraceptive continuation by prompting timely reinjection.^16^ At enrolment, participants provided a contact phone number (either a personal phone or that of a friend, neighbour or relative) to facilitate scheduling the baseline survey. On phone contact, participants were given the option of participating in the follow-up survey by phone or in their preferred location within the facility catchment area. Interviews were conducted within a time window of 15–18 elapsed weeks after method initiation.

The enrolment survey captured sociodemographic and household economic information; reproductive and contraceptive history; relationship status, decision-making power and dynamics; content, person-centeredness and satisfaction with FP services received on the date of enrolment and fertility and contraceptive intentions, including contraceptive agency and mental health. The follow-up study captured detailed information on contraceptive use dynamics over the period since enrolment, including timing of method initiation, discontinuation and method switching; method satisfaction and experience of and care-seeking for contraceptive-induced side effects; current pregnancy status; sexual behaviour; contraceptive preferences and intentions and sexual and reproductive empowerment.

### Ascertainment and definitions of key variables

#### Preference-aligned fertility management

PFM, the primary variable of interest, was ascertained by capturing information on current desire to use contraception and current actual contraceptive use. Current desire to use contraception was captured using a single self-reported item, “Do you currently want to be using a method to delay or avoid pregnancy?”, with response options, ‘yes’, ‘no’ or ‘do not know’. Current contraceptive use was captured by a series of questions: all participants were asked, “Since that visit [at study enrolment], did you start using [ENROLMENT METHOD]?” If the participant affirmed initiation of the enrolment method, they were asked, “Are you still using [ENROLMENT METHOD]?” Participants who reporting never initiating or not currently using the method obtained at enrolment were asked, “Are you (or your partner) currently doing something or using any method to delay or avoid getting pregnant?” Questions about current contraceptive use allowed participants to respond with ‘yes’, ‘no’ or ‘sometimes’. Sometimes users were asked a follow-up question, “You said that you sometimes use a method to prevent pregnancy. Are there times you want to use a method(s) to prevent pregnancy but are not able to?”

PFM was defined as a binary variable based on concordance between reported current desire and actual use of contraception (table 1). Our primary definition of PFM is modified from that proposed by Holt *et al* 14 in several respects. First, Holt *et al* propose ascertainment of concordance between current and desired use by asking about desire to use each specific method in current use. We simplified this definition by asking about desire to use contraception without reference to a specific method. Furthermore, Holt *et al* propose asking current sometimes users if they are able to use the method every time they want to for each episodic method reported. We simplified this follow-up question to ask about their ability to use contraception each time desired, without reference to a specific method. As proposed by Holt *et al*, sometimes users who reported times when they wished to use contraception but were unable to were classified as not practising PFM. A detailed comparison of the definition proposed by Holt *et al* to our modified ascertainment is available in the online supplemental table S1.

10.1136/bmjgh-2023-013902.supp1 Supplementary data

**Table 1:** Classification of PFM (adapted from Holt *et al* 14)

Panel A. PFM (modified)*				
		**Wants to use (any) contraception**	**Does not want to use (any) contraception**	**Does not know whether wants to use**
Current use of (any) contraception		PFM	No PFM	PFM
Current non-use		No PFM	PFM	PFM
Current sometimes use		PFM^†^	No PFM	PFM^†^
Panel B. Satisfaction-adjusted PFM*				
		**Wants to use (any) contraception**	**Does not want to use (any) contraception**	**Does not know whether wants to use**
Current use of (any) contraception	Satisfied with primary method	PFM	No PFM	PFM
	Dissatisfied with primary method	No PFM		No PFM
Current non-use		No PFM	PFM	PFM
Current sometimes use	Satisfied with primary method	PFM^†^	No PFM	PFM^†^
	Dissatisfied with primary method	No PFM		No PFM

Green cells indicate classification as practising PFM, orange cells not practising PFM. Tables created by the authors and adapted from the original definition of PFM proposed by Holt *et al*.

*While we label this measure PFM, it is modified from the definition of PFM proposed by Holt *et al*, primarily in that we assess concordance between desired and actual use of any contraceptive method (without reference to a specific method), rather than desired use of the method actually being used.

†Report currently wanting to use (any) contraception, current/current sometimes use of any contraception, and report that there are no times that they want to, but cannot, use contraception (without reference to a specific method).

PFM, preference-aligned fertility management.

#### Satisfaction-adjusted PFM

We additionally constructed a new version of PFM, which we call ‘satisfaction-adjusted PFM’. Satisfaction-adjusted PFM is calculated using the variables above plus one additional question, administered only to current users and current sometimes users: “On a scale of 0 to 10, where 0 is completely unsatisfied and 10 is completely satisfied, how satisfied are you using [CURRENT METHOD] to delay or avoid pregnancy?” For participants reporting use of more than one method, current method was defined as the primary method as reported by the participant. Based on standard scoring criteria used for the Net Promoter Score, we classify participants as having method satisfaction for scores of 7–10 and as having method dissatisfaction for scores of 0–6.^17^ We then classify all current users and current sometimes users who report method dissatisfaction as not practising satisfaction-adjusted PFM.

### Statistical analysis

We report descriptive summaries of contraceptive use, method satisfaction, PFM and satisfaction-adjusted PFM using appropriate descriptive statistics (counts and percentages for binary and categorical variables; medians and interquartile ranges for continuous variables). We tested for between-group differences in baseline sociodemographic characteristics and contraceptive characteristics (at both baseline and 3.5-month survey) by PFM using χ^2^ tests for categorical variables and Wilcoxon rank-sum tests for continuous variables. For all analyses, we use a complete case approach without imputation of missing data.

## Results

Among 1103 enrolled study participants, 96% (n=1057) completed the first follow-up survey at 3.5 months postmethod initiation. One participant declined to confirm her current contraceptive use, resulting in a final analysis sample of 1056 girls. Study participants in the analysis sample were primarily 18 (n=396, 38%) and 19 (n=515, 49%) years old, with two-thirds (68%) residing in Kaduna State and 85% Muslim (table 2). One-fourth of participants (n=275/1056) reported currently being in school. A median of 1 (IQR: 1, 2) prior pregnancy and prior birth were reported, with one-fifth (n=208/1056; 20%) reporting a history of contraceptive use prior to study enrolment. Most participants (n=925/1056; 88%) reported having sex daily or several times a week over the past 3-month period. Nearly all participants reported future intentions to have a (or another) child, although most (n=593/1056; 56%) reported a desire to delay having a child for 3–5 years.

**Table 2:** Baseline characteristics of the participants in the analysis sample (n=1056)

	N (%)
Age (median, IQR)	18 (18, 19)
15	17 (2)
16	29 (3)
17	99 (9)
18	396 (38)
19	515 (49)
State	
Kaduna	715 (68)
Nasarawa	341 (32)
Religious affiliation	
Christianity	158 (15)
Islam	898 (85)
Currently in school	275 (26)
Prior use of modern contraception	208 (20)
Number of pregnancies (median, IQR)	1 (1, 2)
Number of births (median, IQR)	1 (1, 2)
Frequency of sex in past 3 months	
Many times a week	358 (34)
A few times a week	567 (54)
A few times every month	97 (9)
Rarely or not at all	27 (3)
Refused	7 (0.7)
Plan to have future child in	
<1 year	49 (5)
1–2 years	346 (33)
3–5 years	593 (56)
5+ years	59 (6)
Never	1 (0.1)
Do not know	8 (0.8)

Table presents original analyses conducted and created by the authors.

IQR, Interquartile Range.

Ninety-five per cent (n=1000/1056) of participants reported currently wanting to use contraception; 5% (n=53) stated not wanting to use contraception, while 0.3% (n=3) were unsure about their desire to use contraception (table 3). Among those wanting contraceptive use, nearly all (n=994/1000) were currently using (n=1019) or sometimes using (n=7) contraception. Among participants reporting sometimes using contraception, none reported that there were times that they wished to use contraception but were unable to. Among the 53 participants reporting that they did not currently want to be using contraception, more than half (n=30/53; 57%) were currently using a contraceptive method. Overall, 97% (n=1020/1056) of study participants were practising PFM at 3.5 months follow-up (table 4). Among participants practising PFM, 98% (n=996/1056) both wanted to (or were unsure of their desire) and were using contraception, while 2% (n=24) neither wanted nor were using contraception. Among participants not practising PFM (n=36), most (83%) were using contraception but did not want to be, while the remaining 17% (n=6) wanted but were not currently using contraception.

**Table 3:** Current contraceptive use, desired use and PFM in the study cohort at 3.5 months

Panel A. PFM (modified)*				
		**Wants to use (any) contraception**	**Does not want to use (any) contraception**	**Does not know whether wants to use**
Current use of (any) contraception		988 (99%)	30 (57%)	1 (33%)
Current non-use		6 (0.6%)	23 (43%)	1 (33%)
Current sometimes use		6 (0.6%)	0 (0%)	1 (33%)
Panel B. Satisfaction-adjusted PFM*				
		**Wants to use (any) contraception**	**Does not want to use (any) contraception**	**Does not know whether wants to use**
Current use of (any) contraception	Satisfied with primary method	954 (95%)	29 (55%)	1 (33%)
	Dissatisfied with primary method	34 (3%)	1 (2%)	0 (0%)
Current non-use		6 (0.6%)	23 (43%)	1 (33%)
Current sometimes use	Satisfied with primary method	6 (0.6%)	0 (0%)	1 (33%)
	Dissatisfied with primary method	0 (0%)		0 (0%)

Green cells indicate classification as practising PFM, orange cells not practising PFM. All percentages in the table are column percentages. Table presents original analyses conducted and created by the authors.

*While we label this measure PFM, it is modified from the definition of PFM proposed by Holt *et al*, primarily in that we assess concordance between desired and actual use of any contraceptive method (without reference to a specific method), rather than desired use of the method actually being used.

PFM, preference-aligned fertility management.

**Table 4:** Cross-tabulation of current contraceptive use and PFM

Panel A. PFM (modified)*			
	**No PFM**	**PFM**	**Total**
	**N (%)**	**N (%)**	**N (%)**
By contraceptive use			
Current use/sometimes use	30 (83)	996 (98)	1026 (97)
Current non-use	6 (17)	24 (2)	30 (3)
Total	36 (3)	1020 (97)	1056 (100)
Panel B. Satisfaction-adjusted PFM*			
	**No PFM**	**PFM**	**Total**
	**N (%)**	**N (%)**	**N (%)**
By contraceptive use			
Current use/sometimes use	64 (91)	962 (98)	1026 (97)
Current non-use	6 (9)	24 (2)	30 (2)
Total	70 (7)	986 (93)	1056 (100)

All percentages represent column percentages with the exception of ‘total’ rows, where percentages are row percentages. Table presents original analyses conducted and created by the authors.

*While we label this measure PFM, it is modified from the definition of PFM proposed by Holt *et al,* 14 primarily in that we assess concordance between desired and actual use of *any* contraceptive method (without reference to a specific method), rather than desired use of the method actually being used.

PFM, preference-aligned fertility management.

**Figure 1:**
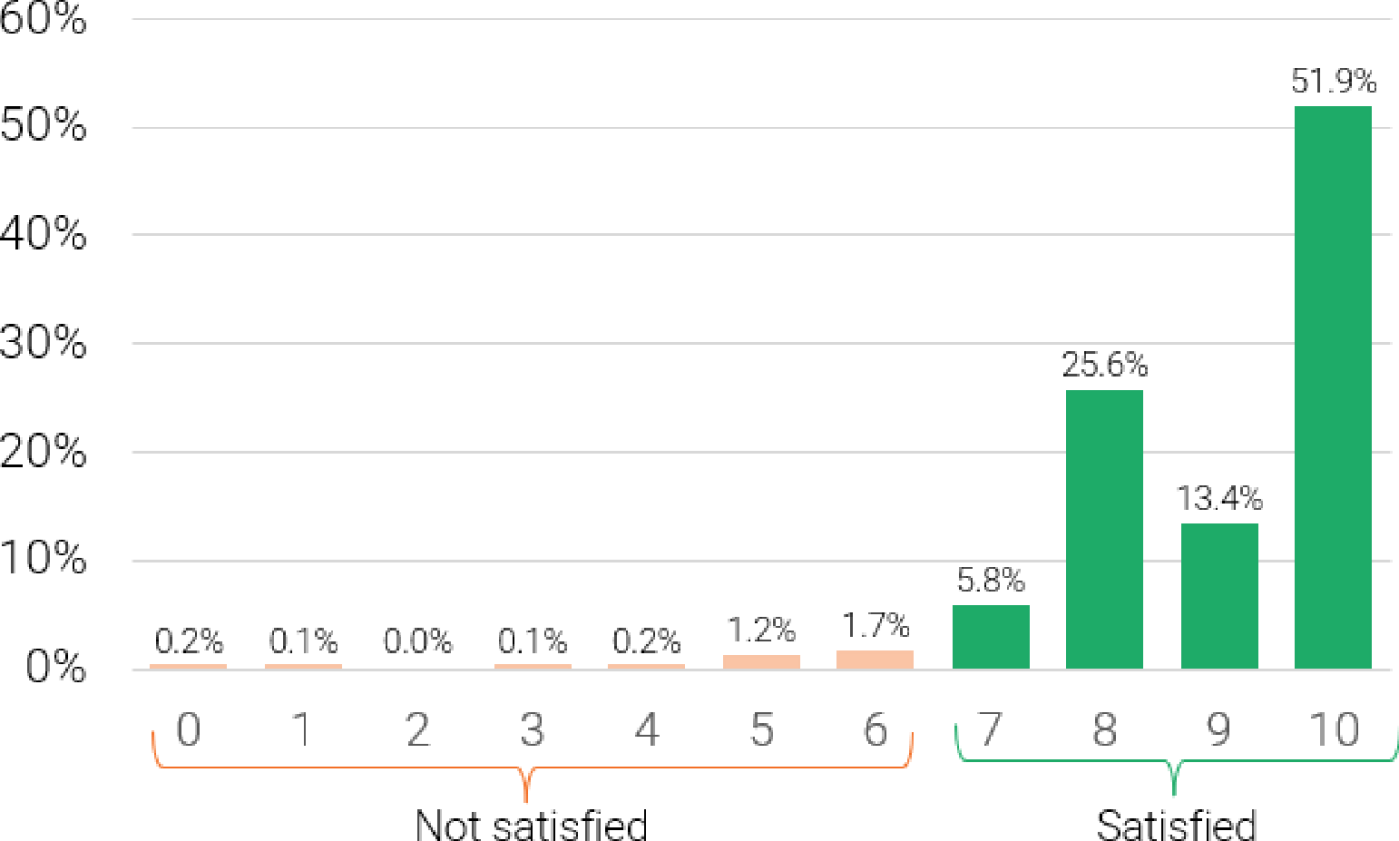
Level of satisfaction with current contraceptive method, among current users and current ‘sometimes’ users at 3.5 months post-initiation (n=1026).

Most current and sometimes users (97%) reported some level of method satisfaction, with 31% reporting scores of 7 or 8 and 65% reporting scores of 9 or 10 (out of a maximum of 10 indicating complete satisfaction) (figure 1). Only 3% (n=35) reported method dissatisfaction, classified as scores of 0 (completely unsatisfied) to 6. Incorporating method satisfaction into the PFM metric to create a satisfaction-adjusted PFM resulted in the reclassification of 34 participants from practising to not practising PFM, for a total prevalence of satisfaction-adjusted PFM of 93%.

Among current and current ‘sometimes’ users at 3.5 months, we find evidence of statistically significant differences in the distribution of current method type by both PFM and satisfaction-adjusted PFM (table 5). The relative distribution of LARC method use was higher (33%, n=10/36) among participants not practising PFM relative to those practising PFM (21%, n=209/1020), although this difference was not statistically significant (p=0.10). No participants using implants in either group reported an unsuccessful attempt to have their current implant removed. Method satisfaction was also lower among participants not practising PFM (median: 8, IQR: 8, 10) relative to those practising PFM (median: 10, IQR: 8, 10), although this difference failed to reach statistical significance at conventional levels (p=0.052). By definition, method satisfaction was lower among current/sometimes users not practising satisfaction-adjusted PFM (median: 6, IQR: 5, 8) compared with those practising satisfaction-adjusted PFM (median: 10, IQR: 8, 10) (p<0.001). We also observed statistically significant differences in the distribution of state in which the enrolment health facility was located and religion by both PFM and satisfaction-adjusted PFM. Respondents practising PFM at 3.5 months were more likely to have been enrolled at a health facility located in Kaduna State versus Nasarawa (p<0.001 for PFM and p=0.01 for satisfaction-adjusted PFM) and to report being Muslim versus Christian (p<0.001 for PFM and satisfaction-adjusted PFM). Older age (p=0.04) and school attendance (p=0.01) were associated with practising PFM but not satisfaction-adjusted PFM, while greater number of births was associated with practising satisfaction-adjusted PFM (p=0.03) but failed to reach satistical significance for PFM (p=0.09).

**Table 5:** Sociodemographic and contraceptive use correlates of PFM

	PFM (modified)*			Satisfaction-adjusted PFM		
	No	Yes	P value	No	Yes	P value
	N (%)	N (%)		N (%)	N (%)	
Age (median, IQR)	18 (18, 19)	18 (18, 19)	0.04†	18 (18, 19)	18 (18, 19)	0.57†
State						
Kaduna	12 (33)	703 (69)	<0.001	38 (54)	677 (69)	0.01
Nasarawa	24 (67)	317 (31)		32 (46)	309 (31)	
Religion						
Christianity	19 (53)	139 (14)	<0.001	24 (34)	134 (14)	<0.001
Islam	17 (47)	881 (86)		46 (66)	852 (86)	
Currently in school	3 (8)	272 (27)	0.01	15 (21)	260 (26)	0.36
Frequency of sex in past 3 months						
Many times a week	18 (50)	340 (33)	0.25	27 (39)	331 (34)	0.09
Few times a week	13 (36)	554 (54)		29 (41)	538 (55)	
A few times every month	4 (11)	93 (9)		10 (14)	87 (9)	
Rarely or not at all	1 (3)	26 (3)		4 (6)	23 (2)	
Refused	0 (0)	7 (0.7)		0 (0)	7 (0.7)	
Number of pregnancies (median, IQR)	1 (1, 2)	1 (1, 2)	0.68†	1 (1, 2)	1 (1, 2)	0.87†
Number of births (median, IQR)	1 (0.5, 1.5)	1 (1, 2)	0.09†	1 (1, 2)	1 (1, 2)	0.026†
Plan to have a/nother child in						
<1 year	3 (8)	46 (5)	0.32	8 (11)	41 (4)	0.13
1–2 years	14 (39)	332 (33)		21 (30)	325 (33)	
3–5 years	18 (50)	575 (56)		37 (53)	556 (56)	
5+ years	0 (0)	59 (6)		3 (4)	56 (6)	
Never	0 (0)	1 (0.1)		0 (0)	1 (0.1)	
Do not know	1 (3)	7 (0.7)		1 (1)	7 (0.7)	
Prior use of contraception at study enrolment	7 (19)	201 (20)	0.97	15 (21)	193 (20)	0.71
Contraceptive method at study enrolment						
IUD	0 (0)	11 (1)	0.61	2 (3)	9 (0.9)	0.33
Implant	12 (33)	201 (20)		21 (30)	192 (19)	
Injectable	17 (47)	610 (60)		34 (49)	593 (60)	
OCPs	6 (17)	177 (17)		12 (17)	171 (17)	
ECP	0 (0)	1 (0.1)		0 (0)	1 (0.1)	
Male condom	1 (3)	13 (1)		1 (1)	13 (1)	
Female condom	0 (0)	6 (0.6)		0 (0)	6 (0.6)	
Other	0 (0)	1 (0.1)		0 (0)	1 (0.1)	
Current method use at 15-week follow-up	30 (83)	996 (98)	<0.001	64 (91)	962 (98)	0.003
Current method type, among 15-week current users						
IUD	0 (0)	9 (0.9)	<0.001	0 (0)	9 (0.9)	<0.001
Implant	10 (33)	200 (20)		18 (28)	192 (20)	
Injectable	16 (53)	620 (62)		30 (47)	606 (63)	
Oral contraceptive pills	1 (3)	152 (15)		10 (16)	143 (15)	
Emergency contraceptive pills	1 (3)	0 (0)		1 (2)	0 (1)	
Male condom	1 (3)	11 (1)		3 (5)	9 (0.9)	
Female condom	0 (0)	3 (0.3)		0 (0)	3 (0.3)	
Withdrawal	1 (3)	1 (0.1)		2 (3)	0 (0)	
Use of LARC method, among 15-week current users	10 (33)	209 (21)	0.10	18 (28)	201 (21)	0.17
Method satisfaction, among 15-week current users (median, IQR)	8 (8,10)	10 (8, 10)	0.05†	6 (5,8)	10 (8, 10)	<0.001†

The full analysis sample comprises 36 participants with no PFM and 1020 participants with PFM; analyses among 15-week current contraceptive users include 30 participants not practicing PFM and 996 practicing PFM. Table presents original analyses conducted and created by the authors.

*While we label this measure PFM, it is modified from the definition of PFM proposed by Holt *et al*,^14^ primarily in that we assess concordance between desired and actual use of any contraceptive method (without reference to a specific method), rather than desired use of the method actually being used.

†Wilcoxon rank-sum test; all other p values estimated using χ^2^ tests.

IUD, intrauterine device; LARC, long-acting reversible contraception (implants and IUD); PFM, preference-aligned fertility management.

## Discussion

We fielded modified versions of the newly proposed PFM measure within an ongoing cohort study of Nigerian married adolescent girls. As girls were enrolled in the cohort at the point of modern contraceptive method initiation and assessed for PFM after just 3.5 months, we were not surprised to find that prevalence of PFM and modern contraceptive use was high. However, although mCP and PFM were each estimated at 97%, the two measures define contraceptive ‘success’ differently—as contraceptive use and self-perceived contraceptive autonomy, respectively. Despite contraceptive method initiation just over 3 months prior, 5% of the cohort reported not wanting to use contraception; among these, more than half were currently using a contraceptive method despite not wanting to. An additional 3% of the cohort reported being dissatisfied with their current method. These girls would all be considered programmatic ‘successes’ using contraceptive utilisation-focused end points. PFM classifies this discordance as a lack of self-perceived contraceptive autonomy. A shift in measurement from use-focused measures to PFM would therefore have important implications for programming: if integrated within routine programme monitoring and evaluation, PFM could be used to identify non-autonomous contraceptive use within the context of specific programmes and to more effectively direct targeted support (whether to identify and remove barriers to removal or access, or to support method stopping, switching or initiation) to participants who are not currently able to act on their contraceptive preferences.

The primary reasons for discordance between contraceptive behaviours and desires in this cohort are unclear. Between-group differences in sociodemographic characteristics, such as State and respondent religion, should be interpreted with caution, as the facility-based sampling approach does not allow for representativeness within these groups. While we observed that a higher proportion of current contraceptive users not practising PFM were LARC users, relative to current contraceptive users practising PFM, discordance between contraceptive use and desire in this group does not appear to be driven by failed attempts at LARC removal. However, it is possible that barriers to LARC removal such as high price or resistant providers (well documented in other studies^5 6 18^) may have discouraged current implant users with no desire to use contraception from even attempting removal. We did not have the opportunity to conduct cognitive interviews to understand women’s understanding and interpretation of these questions. In future research, it will be important to conduct cognitive interviewing to clarify question wording and to layer qualitative and quantitative follow-up questions to better understand the reasons for discordance between preferences and use in both directions. This is critical for elucidating programmatically actionable information to address barriers to perceived contraceptive autonomy. We did not have the opportunity to conduct cognitive interviewing to assess comprehension of the questions.

PFM is a measure of self-perceived contraceptive autonomy that captures whether an individual’s stated preferences align with their current behaviours. Part of the PFM measure’s appeal is its relative simplicity and parsimony—ascertainment requires very few additional questions to standard FP survey instruments. This greatly increases the likelihood of its scalability across diverse contexts and within multicountry survey instruments. The measure also has the advantage of capturing women’s self-perceived contraceptive autonomy, rather than prescribing factors that must be in place for autonomous contraceptive practice to occur.^19^ On the other hand, the PFM measure does not provide detailed information on whether enabling conditions (or perhaps even requirements) for ‘full, free and informed’^1^ contraceptive choices are in place, such as comprehensive and accurate knowledge of contraception within an affordable, acceptable and non-coercive enabling contraceptive environment. Bullington *et al* have discussed the relative advantages of ‘self-perceived’ versus ‘researcher-ascribed’ measures in assessing constructs such as informed contraceptive choice, noting that these differing measurement approaches each capture important but distinct information.^19^ As a result, we envision the PFM measure as complementary to Senderowicz’ Contraceptive Autonomy Scale^1 20^ and other rights-based FP measures.

From a measurement perspective, we found that PFM and mCP identify meaningfully different subpopulations of the cohort. This is an important finding, as it is unclear to what extent factors such as post hoc rationalisation might influence women to report concordance between actual use and desired use. While it is true that self-reporting biases such as social desirability bias or post hoc rationalisation may bias estimates of PFM upwards, we nevertheless observed a notable degree of discordance between desires and use in this sample of adolescent girls.

We also considered the implications of method dissatisfaction among current users in a satisfaction-adjusted definition of PFM. Relatively little is known about method dissatisfaction among current users, as demographic and health surveys typically only ask women not using or recently discontinuing contraception about method-related problems. However, a growing body of literature suggests that many women continue using contraceptive methods despite experience of adverse side effects and substantial levels of dissatisfaction with the method.^21–23^ Using standard scoring guidance for the Net Promoter Score, a widespread measure of client satisfaction developed for commercial applications, nearly one-third of our sample could be considered ‘passive’ in terms of their method satisfaction, while another 3.5% would be classified as actively dissatisfied ‘detractors’ of their contraceptive method. Reclassifying this group of dissatisfied users more than doubled the percentage of participants who were considered not to be practising contraception in line with their preferences: using a modified version of the measure proposed by Holt *et al*, 97% of participants were practising PFM; this percentage decreased to 93% using a measure that incorporates method dissatisfaction, which we are calling satisfaction-adjusted PFM.

This study has several strengths. This is the first report, to our knowledge, describing the collection and results of a newly proposed and promising measure of self-perceived contraceptive autonomy, PFM. Tested within an ongoing cohort study, our report of a point prevalence at 3.5 months postcontraceptive initiation is unlikely to be biased due to attrition given high retention in the cohort. Finally, we demonstrate the viability of PFM as an end point for longitudinal studies of contraceptive use dynamics, presenting a rights-based alternative to contraceptive continuation.

Our approach also has several limitations. We did not measure PFM at baseline, so it is not clear the extent to which PFM changed over the 15-week period between enrolment and the first follow-up survey reported here. We intend to capture PFM across multiple timepoints in this ongoing cohort, which will allow us to assess PFM’s stability over time. We also simplified the primary definition of PFM relative to the approach proposed by Holt *et al*; this was in part because we fielded a preliminary, prepublication version of the PFM instrument which was later revised by Holt *et al*, and in part due to intentional simplification of the questions to reduce survey burden on participants. The latter was particularly important to us, as participants participated in the follow-up survey by phone, making a lengthy questionnaire impractical. As a result, we are unable to assess how PFM ascertainment differs between our version and that proposed by Holt *et al*. Specifically, we did not ask women about their desire to use *specific methods* of contraception. Ensuring that women’s method-specific preferences are met is critical, and an overlooked aspect of current contraceptive research.^24^ Future studies would benefit from assessing the advantages and disadvantages of asking women about their desired use of specific methods, relative to the simplicity of asking about their blanket desire to use contraception (as was field-tested in this study). While capturing method-specific preferences and/or method satisfaction may be optimal, inclusion should nevertheless be weighed against the advantages in terms of usability and interpretability of a simpler measure. Finally, our study was conducted in a specific population of participants in a programme designed to provide adolescent responsive contraceptive services and care to married adolescent girls in Northern Nigeria. As such, our results have limited generalisability beyond the study setting. We recommend additional piloting in the context of a population-based study as a next step.

## Conclusion

As the FP field struggles to agree on a new direction for evaluating FP programme performance, the concepts of contraceptive autonomy, fulfilment of rights and universal access are taking centre stage. PFM is a pragmatic measure of self-perceived contraceptive autonomy that could provide actionable data at the programme, subnational, national and global levels; and that could be feasibly incorporated into existing performance monitoring frameworks. As such, it takes us one step closer to our collective goal of achieving universal access to rights-based sexual and reproductive healthcare.

10.1136/bmjgh-2023-013902.supp2 Supplementary data

## Data Availability

Data are available on reasonable request. The data that support the findings of this study comprise a subset of the data collected as part of an ongoing cohort study and therefore are not yet publicly available. Data may be made available on a case-by-case basis on reasonable request to the corresponding author.
