# Supplementary material for: Preference-aligned fertility management among married adolescent girls in Northern Nigeria: assessing a new measure of contraceptive autonomy
Source: BMJ Glob Health. 2024 May 16;9(5):e013902. doi: 10.1136/bmjgh-2023-013902 (PMC11103226; doi:10.1136/bmjgh-2023-013902)
Supplement: Supplementary data [file bmjgh-2023-013902supp001.pdf]

Preference-aligned fertility management among married adolescent girls in Northern Nigeria: Assessing a new measure of contraceptive autonomy

Supplemental Materials

Table S1. Variables used to ascertain preference-aligned fertility management, comparing the implemented approach to that proposed by Holt et al. (2023)

	PFM*	Response Options	# Questions	PFM (Modified by Rothschild et al.)	# Questions
Current FP desire	Do you currently want to be using any method to avoid pregnancy – that is, to do something to keep it from happening?	No<br>Yes<br>Don't know	1	Do you currently want to be using a method to delay or avoid pregnancy?	1
Current FP desire for specific method	[For each method reported]: Do you want to be using this method right now?	No<br>Yes<br>Don't know	1 – total # reported methods	*Not asked*	
Current FP use	Are you currently using any method to avoid pregnancy?	No<br>Yes<br>Sometimes*	1	Are you (or your partner) currently doing something or using any method to delay or avoid getting pregnant?*	1
Current sometimes use	[For each episodic method reported]: You said that you sometimes use [METHOD] to prevent pregnancy. During the times when you want to use this method to prevent pregnancy, are there times you want to use this but are not able to?	No<br>Yes	1 – total # reported episodic methods	You said that you sometimes use a method to prevent pregnancy. Are there times you want to use a method(s) to prevent pregnancy but are not able to?	1

* Variable ascertainment proposed by Holt et al. (2023) in Holt et al. Preference-aligned fertility management as a person-centered alternative to contraceptive use-focused measures. Stud Fam Plann. 2023; https://doi.org/10.1111/sifp.12228
