# Supplementary material for: Preference-aligned fertility management among married adolescent girls in Northern Nigeria: assessing a new measure of contraceptive autonomy
Source: BMJ Glob Health. 2024 May 16;9(5):e013902. doi: 10.1136/bmjgh-2023-013902 (PMC11103226; doi:10.1136/bmjgh-2023-013902)
Supplement: Supplementary data [file bmjgh-2023-013902supp002.pdf]

Online Supplement S2 – Reflexivity Statement

1. How does this study address local research and policy priorities?

This study describes the self-identified contraceptive preferences and needs of married adolescent girls receiving contraceptive services from a subset of public health facilities supported by the Matasa Matan Arewa (MMA) program. The MMA program is implemented by Society for Family Health-Nigeria (SFH) and the Nigerian Federal Ministry of Health (FMOH) as part of the multi-country Adolescents 360 program led by Population Services International (PSI). With the support of the Nigerian FMOH, the MMA program is being scaled to support delivery of adolescent-responsive sexual and reproductive health (SRH) services within the Nigerian public health system. A key component of MMA's learning and evidence approach is to generate evidence to be used for adaptive program management and iterative improvement. This study describes experiences of program participants, and local dissemination of these findings will be used to inform program adaptations for implementation by SFH and the FMOH. As such, the study contributes to evidence-based SRH program implementation and adolescent SRH policy within Nigeria's public health sector.

2. How were local researchers involved in study design?

The analysis presented in this paper uses data collected as part of a parent study which has the primary aim of exploring contraceptive use dynamics and experiences among married adolescent girls initiating contraception at MMA-supported health facilities. The research team comprises researchers from Population Services International and Society for Family Health-Nigeria. These investigators were involved in design and protocol development. SFH and the FMOH are responsible for implementation of the MMA program. SFH collected the study data, with supervision and support from PSI.

3. How has funding been used to support the local research team?

Adolescent 360 (A360) project funding has been used to support SFH's implementation – in partnership with the FMOH – of the MMA project. In addition, project funding dedicated for evidence and learning was used to conduct this research. Direct support was provided to the SFH team to conduct primary data collection (including participant recruitment and baseline and follow-up surveying, as well as quality assurance activities and data management). Supervision and support for field team recruitment, training, data collection, and data quality assurance was provided by the PSI team, who worked closely with SFH to conduct this research. A360 project funding was used to strengthen the local research team's capacity for high-quality primary data collection and field team management; data management and cleaning procedures; and will provide SFH research team members with opportunities for first authorship and co-authorship as desired in planned research outputs from the parent study.

4. How are research staff who conducted data collection acknowledged?

SFH and PSI co-investigators who were involved in direct field team management and data collection (AB, RO, SCI), and who supervised data collection and data quality assurance (CWR, JN, AM) are included as authors. The full data collection team are also acknowledged in the paper in the Acknowledgments section.

5. Do all members of the research partnership have access to study data?

As members of the research team, SFH and PSI investigators named on the protocol have access to the study data and continue to actively collaborate to define additional research products from the study.

6. How was data used to develop analytical skills within the partnership?

PSI investigators worked with SFH investigators to strengthen capacity for quantitative data management and cleaning, which were conducted collaboratively. This involved frequent calls between the PSI and SFH research team members to discuss data quality and management.

7. How have research partners collaborated in interpreting study data?

Authors CWR, JN, and AM worked closely to design this specific study, analytic approach, and interpretation of findings. Findings were shared along with a first draft of the manuscript with SFH co-authors AB, RO, and SCI, who provided detailed feedback on the findings and contributed additional contextual information for their appropriate interpretation.

8. How were research partners supported to develop writing skills?

The first draft of the manuscript was written by the first author (CWR). Research partners were given opportunity to revise the document directly or to suggest revisions via comments, which were later incorporated by the first author. While this paper did not directly support any further development of writing skills within the research partnership, the broader evidence and learning activities of the A360 program include activities aimed to develop research writing skills within the research partnership, including commissioning and supporting development of knowledge and evidence products and opportunities for first-authored publications and conference abstracts.

9. How will research products be shared to address local needs?

The research team, along with implementing partners at the Nigerian FMOH, have regularly disseminated evidence and learning products from the A360 project through dissemination events at the national and local governing authority (LGA) levels. A major dissemination platform of the A360 program in half-yearly “pause-and-reflect” workshops, which bring together program and government stakeholders to review evidence and design solutions collectively based on program evidence and learning. Findings from this study, which is a part of the broader A360 evidence and learning agenda, will be similarly shared through planned dissemination events and project reports.

10. How is the leadership, contribution and ownership of this work by LMIC researchers recognised within the authorship?

Along with the first author CWR, LMIC-based researchers JN and AM were closely involved in refining the study aims and supporting data analysis and interpretation of findings. AM is recognized as the senior author of this paper. We have also included LMIC authors AB, RO, and SCI as co-authors, given their leadership in study design, primary data collection, and contextualization and interpretation of findings. We acknowledge that the first author (CWR) is based in a high-income country. This is due to the first author’s involvement in global measurement activities, including design and testing of novel, rights-based family planning measures in a variety of global settings.

11. **How have early career researchers across the partnership been included within the authorship team?**

Most of the authorship team is comprised of early career researchers and/or senior programmatic staff members. A senior researcher (KT) provided mentorship to the first author (CWR) to advise analytic approaches and assist in interpretation of findings.

12. How has gender balance been addressed within the authorship?

Four authors are female (CWR, RO, SCI, KT) and three authors are male (AB, JN, AM).

13. How has the project contributed to training of LMIC researchers?

All authors are based in LMIC except for two (CWR, KT). AM is the senior research lead for the A360 program; along with CWR and JN, AM provided in-depth research training and supervision to LMIC researchers at SFH who were involved in primary data collection, data management, and cleaning. Training of these researchers included modules on general research topics (e.g., human subjects research), standard operating procedures, and issues specific to the prospective cohort design. In addition, JN and AM provided structured support to LMIC researchers at SFH to strengthen capacity for reproducible research and best practices in data management.

14. How has the project contributed to improvements in local infrastructure?

As mentioned previously, this study was conducted as part of the evidence and learning agenda for the A360 program, which is implemented locally in Northern Nigeria as the MMA program. The explicit purpose of this learning agenda and the broader scope of research conducted under A360 is to provide actionable evidence for adaptive management and program improvement in the delivery of adolescent-responsive contraceptive and other SRH services within Nigeria's public sector. A360's learning products have supported the project to secure government buy-in for scaled implementation in Nigeria's public health system, thereby expanding delivery of adolescent-friendly SRH services.

15. What safeguarding procedures were used to protect local study participants and researchers?

Ethical approval for this research study was obtained both from the Population Services International Research Ethics Board as well as the Nigerian Health Research and Ethics Committee. The senior author (AM) and JN worked closely with Society for Family Health-Nigeria researchers to ensure data quality, including backcheck mechanisms with opportunities for study participants to share any concerns or problems with the study team. Regular refresher trainings and field team meetings with SFH and PSI researchers ensured that the study was implemented per the approved protocol and in line with ethical guidelines and best practices. In addition, the research team worked closely with the Nigeria-based program team and implementing partners to assess and manage security issues in study sites to minimize possible risks to the study participants and researchers and other staff members.
